# Sudden Onset, Fixed Dystonia and Acute Peripheral Trauma as Diagnostic Clues for Functional Dystonia

**DOI:** 10.1002/mdc3.13322

**Published:** 2021-09-10

**Authors:** Tommaso Ercoli, Giovanni Defazio, Christian Geroin, Enrico Marcuzzo, Giovanni Fabbrini, Francesco Bono, Alessandro Mechelli, Roberto Ceravolo, Luigi Michele Romito, Alberto Albanese, Antonio Pisani, Maurizio Zibetti, Maria Concetta Altavista, Luca Maderna, Martina Petracca, Paolo Girlanda, Marcello Mario Mascia, Alfredo Berardelli, Michele Tinazzi, for the Italian Registry of Functional Motor Disorders Study Group, the Italian Registry of Adult Dystonia Study Group

**Affiliations:** ^1^ Department of Medical Sciences and Public Health University of Cagliari Cagliari Italy; ^2^ Neurology Unit, Movement Disorders Division, Department of Neurosciences, Biomedicine and Movement Sciences University of Verona Verona Italy; ^3^ Department of Human Neurosciences Sapienza University of Rome Rome Italy; ^4^ IRCCS Neuromed Pozzilli Italy; ^5^ Botulinum Toxin Center, Neurology Unit A.O.U. Mater Domini Catanzaro Italy; ^6^ Neurology Unit, Department of Clinical and Experimental Medicine University of Pisa Pisa Italy; ^7^ Parkinson and Movement Disorders Unit Fondazione IRCCS Istituto Neurologico Carlo Besta Milan Italy; ^8^ Department of Neurology IRCCS Humanitas Research Hospital Rozzano Italy; ^9^ Department of Brain and Behavioral Sciences University of Pavia Pavia Italy; ^10^ IRCCS Mondino Foundation Pavia Italy; ^11^ Department of Neuroscience–Rita Levi Montalcini University of Turin Turin Italy; ^12^ UOC di Neurologia Ospedale San Filippo Neri Rome Italy; ^13^ Department of Neurology‐Stroke Unit and Laboratory of Neurosciences Istituto Auxologico Italiano, IRCCS Milan Italy; ^14^ Fondazione Policlinico Universitario ‘Agostino Gemelli’–IRCCS Rome Italy; ^15^ Department of Clinical and Experimental Medicine University of Messina Messina Italy

**Keywords:** idiopathic dystonia, functional dystonia, sudden onset, fixed dystonia, acute peripheral trauma

## Abstract

**Background:**

The differentiation of functional dystonia from idiopathic dystonia may be clinically challenging.

**Objective:**

To identify clinical features suggestive of functional dystonia to guide physicians to distinguish functional dystonia from idiopathic dystonia.

**Methods:**

Patient data were extracted from the Italian Registry of Functional Motor Disorders and the Italian Registry of Adult Dystonia. Patients with functional and idiopathic dystonia were followed up at the same clinical sites, and they were similar in age and sex.

**Results:**

We identified 113 patients with functional dystonia and 125 with idiopathic dystonia. Sudden onset of dystonia, evidence of fixed dystonia, and acute peripheral trauma before dystonia onset were more frequent in the functional dystonia group. No study variable alone achieved satisfactory sensitivity and specificity, whereas a combination of variables yielded 85% sensitivity and 98% specificity. A diagnostic algorithm was developed to reduce the risk of misclassifying functional dystonia.

**Conclusion:**

Our findings extend the current diagnostic approach to functional dystonia by showing that clinical information about symptom onset, fixed dystonia, and history of peripheral trauma may provide key clues in the diagnosis of functional dystonia.

Inconsistency (ie, changing patterns over time with susceptibility to distraction) and/or incongruence (ie, a clinical picture incompatible with known determined patterns) are clinical features of neurological examination that support clinically definite diagnosis of functional dystonia (FDYT) according with the most recent set of diagnostic criteria proposed by Gupta and Lang.^1^ Demonstrating inconsistency/incongruence may be clinically challenging, however,^2^, ^3^ and straightforward laboratory‐supported criteria for most forms of dystonia are lacking.^4^ The only reliable neurophysiological discriminator between FDYT and idiopathic dystonia (IDYT) proposed to date has been for blepharospasm.^5^

Medical history and clinical features that may reveal some clues to the diagnosis of FDYT include sudden symptom onset, evidence of fixed movement disorder, history of physical trauma, psychiatric diseases, and comorbid functional somatic disorders. Their validity in supporting a diagnosis of FDYT remains to be fully established.

For this study, we compared the frequency of sudden symptom onset, evidence of fixed dystonia, and prior acute peripheral trauma in patients with adult‐onset FDYT and IDYT. We also assessed their sensitivity and specificity either alone or combined. We did not assess psychiatric diseases and comorbid functional somatic disorders because of their high frequency in both FDYT and IDYT.^6^

## Patients and Methods

The study relied on information from the Italian Registry of Functional Motor Disorders (IRFMD) and the Italian Registry of Adult Dystonia (IRAD), 2 multicenter initiatives coordinated by the Italian Academy for the Study of Parkinson's Disease and Other Movement Disorders (Accademia LIMPE‐DISMOV RADAC project) and Fondazione LIMPE. Patients in the IRFMD were referred from 25 Italian centers for movement disorders with a diagnosis of clinically definite functional motor disorders (FMDs) based on Gupta and Lang's diagnostic criteria.^1^ The IRAD includes patients with adult‐onset dystonia from 37 secondary/tertiary referral centers for movement disorders throughout Italy. Diagnosis was made according to published criteria.^7^, ^8^ Core assessment characterizing IRFMD and IRAD has been described in detail elsewhere^9^, ^10^; it comprises demographic, historical, and clinical information on the movement disorder and possible predisposing/precipitating factors.^11^, ^12^, ^13^

Information was collected about dystonia at different body sites (upper and lower limbs, trunk, cervical, cranial among others), year of dystonia onset, and prior peripheral injury (at extracranial body sites). Onset of dystonia can be defined as acute (abrupt with deterioration within a few days or weeks) or slowly progressing.^10^ Peripheral injury had to be severe enough to require medical attention, hospitalization, or surgery. Information about trauma included year and site of the injury. Dystonia was defined as fixed when immobile dystonic postures did not return to the neutral position at rest.^14^ Patients with FDYT from the IRFMD were frequency matched by age and sex with patients with IDYT from the IRAD who were followed up at the same clinics. To include only patients who were idiopathic, tests for Wilson's disease, dopa‐responsive dystonia, and common genetic variants (eg, *TOR1A*, *THAP1*) were performed as appropriate. Patients screening positive on genetic testing were not included in the study sample.^15^

Statistical analysis was performed using the Stata 11 package (StataCorp, College Station, TX) and descriptive and inferential statistics as appropriate (*t* test, chi‐square test, Fisher's exact test). Data are expressed as mean ± standard deviation unless otherwise indicated. Logistic regression models for unequal case‐control ratios were computed to assess the association between history of trauma and case‐control status after adjusting for potentially confounding variables. Statistical significance was set at 0.05. To assess sensitivity and specificity, the gold standard was the diagnosis of FDYT or IDYT made at each site. Sensitivity was defined as the proportion of patients who screened positive among those given a diagnosis of FDYT (true positives/true positives + false negatives). Specificity was the proportion of patients who screened negative among those given a diagnosis of IDYT (true negatives/false positives + true negatives).

## Results

In December 2020, the data on 113 patients with FDYT were extracted from the IRFMD, which included data from 410 patients with functional movement disorders,^13^, ^16^ and 125 patients with IDYT selected among the 1634 patients from the IRAD.^17^ The 2 groups were similar for sex, age, and educational level but differed for disease duration, dystonia distribution, and frequency of focal dystonia, which was more frequent in the patients with IDYT (Table 1). Limb/trunk dystonia was more frequent in the FDYT group, and cranial‐cervical dystonia was more frequent in the IDYT group (Table 1).

**TABLE 1:** Clinical and demographic features of patients with functional and idiopathic dystonia

Clinical/demographic features	Patients with Functional Dystonia (n = 113)	Patients with Idiopathic Dystonia (n = 125)	*P* Value
Women, n (%)	87 (77)	94 (75)	0.4
Age, years, mean ± SD	47.2 ± 14.4	49.4 ± 10.0	0.2
Years of schooling, mean ± SD	12.5 ± 4.1	12.3 ± 3.5	0.7
Age at onset, years, mean ± SD	41.3 ± 14.1	40.2 ± 10.6	0.3
Years of disease duration, mean ± SD	5.9 ± 6.2	9.5 ± 8.0	0.0002
Focal dystonia, n (%)	67 (59.3)	104 (83.2)	<0.0001
Site of dystonia, n (%)			
Upper limbs	52 (46)	24 (19.2)	<0.0001
Lower limbs	43 (38.1)	6 (4.8)	<0.0001
Trunk	22 (19.5)	5 (4)	<0.0001
Cervical	41 (36.3)	84 (67)	<0.0001
Cranial	8 (7.1)	36 (28.8)	<0.0001
Other	11 (9.7)	0	<0.0001
Sudden dystonia onset, n (%)	73 (65)	0	<0.0001
Fixed dystonia, n (%)	56 (49.6)	0	<0.0001
History of peripheral trauma, n (%)	22 (19.5)	3 (2)	<0.0001
Trauma and dystonia in the same body part, n (%)	17 (15)	3 (2)	<0.0001
Months elapsing between trauma in the same body part and dystonia onset, mean ± SD (n)	3.1 ± 6.9 (17)	2.7 ± 3.8 (3)	0.9

SD, standard deviation.

Sudden onset of dystonia, evidence of fixed dystonia, and acute peripheral trauma before dystonia onset were more frequent in the FDYT group (Table 1). This finding was confirmed after limiting the analysis to trauma to the dystonic body part (Table 1) and adjusting for disease duration, age, and sex on logistic regression analysis (adjusted odds ratio, 5.8; 95% confidence interval, 1.6–20.9; *P* = 0.007).

**FIG. 1:**
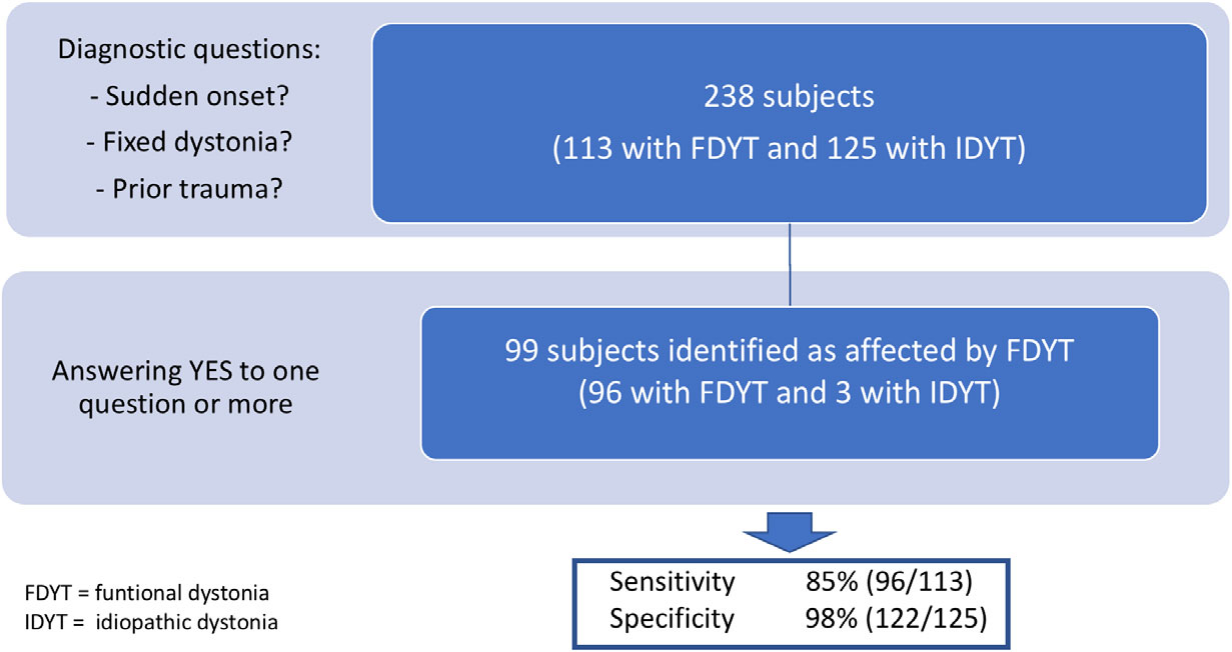
Diagnosing functional dystonia. FDYT, functional dystonia; IDYT, idiopathic dystonia.

No study variable alone reached a satisfactory combination of sensitivity/specificity (sudden onset of dystonia: sensitivity, 65% [73/113] and specificity, 100% [125/125]; fixed dystonia: sensitivity, 50% [56/113] and specificity, 100% [125/125]; prior trauma: sensitivity, 15% [17/113] and specificity, 98% [122/125]). However, screening positive to at least 1 of the 3 variables yielded 85% sensitivity (96/113) and 98% specificity (122/125) (Fig. 1).

## Discussion

Our findings indicate that sudden dystonia onset and fixed dystonia are more likely to occur in FDYT and that acute peripheral trauma may be significantly associated with FDYT.^3^, ^14^ Novel findings indicate that each variable differentiated FDYT from IDYT with 15% to 65% sensitivity, but none of these clinical features alone was crucial for diagnosing FDYT. Nonetheless, each of the 3 variables carried a negligible risk of misclassifying FDYT cases and reached 98% to 100% specificity. Although no study variable alone achieved satisfactory sensitivity and specificity, screening positive to at least 1 of the 3 clinical features can correctly diagnose FDYT in more than 8/10 patients who have the condition (85% sensitivity) and can correctly identify as not having FDYT about 10/10 subjects not affected by the condition (98% specificity). The sensitivity and specificity levels were shared by those reported for a recently published decision tree that classified FDYT using a complex case‐finding procedure based on the serial application of about 7 historical/clinical features and diagnostic confirmation subsequently informed by recognition of incongruence/inconsistence on neurological examination.^18^ At variance with such an approach, however, our algorithm is considerably simpler and based on the combination of only 3 historical/clinical features.

Our study has several strengths. First, the populations of FDYT and IDYT were from multicenter settings and probably representative of the general population of cases with similar demographic/clinical features.^9^, ^10^ The older age at onset of patients with FDYT compared with the other reported cohorts probably reflects the inclusion of only adult‐onset patients in the source registries. The frequency of sudden onset and fixed dystonia in our sample was consistent with previous series, whereas the trauma frequency was lower.^14^ Unlike other studies, however, we limited recall bias by not including patients with mild trauma. Physical injury was more commonly recorded preceding weakness than dystonia.^19^ Second, the standard for comparison was dystonia status based on clinical examination by neurologists applying stringent diagnostic criteria. In addition, both groups of patients with FDYT and IDYT were followed up by the same neurologists at the same center, which provided accuracy in data collection. The low frequency of peripheral trauma to a specific body part in the IDYT group was consistent with the observations of several large controlled studies and demonstrated a negligible effect of peripheral trauma on topographically related IDYT.^20^, ^21^

The present study also has some limitations. Patients participating in the study were diagnosed with clinically definite FMDs according to the Gupta and Lang criteria.^1^ These criteria are largely based on incongruence/inconsistency that may incorporate some of the issues we studied such as sudden onset and fixed dystonia. This may lead to the “circular argument” of diagnosing FMDs with new sets of criteria based on existing criteria. However, our aim was to measure the accuracy of these 3 simple aids alone in recognizing FDYT diagnosed according with the current gold standard, that is, clinical diagnosis established by expert neurologists who relied on several additional aspects of incongruence/inconsistency and positive clinical signs.^22^, ^23^, ^24^ Moreover, because the patients and the physicians involved in the study were from the same country, data from other populations of patients and movement disorder specialists are needed to confirm the present results. Also, because our study focused on dystonia, not all findings may be extensible to other functional movement disorders. Disease duration was significantly lower in the FDYT group, even though the estimated association between FDYT and trauma did not change after adjusting for disease duration. Body distribution of dystonia differed in the FDYT and the IDYT groups, which probably reflects the frequency distribution of dystonia in the general population of adult‐onset cases with functional and idiopathic dystonia. Although we did not match patients with FDYT and IDYT by distribution of dystonia, we were confident that our study variables, in particular sudden onset of dystonia and evidence of fixed dystonia, were probably independent of the body localization of dystonia. Nevertheless, we acknowledge that the higher frequency of limb dystonia in the FDYT group may limit the generalizability of findings to all forms of FDYT.

In conclusion, our findings extend the current diagnostic approach to FDYT by showing that clinical information about symptom onset, fixed dystonia, and previous peripheral trauma may provide key clues for diagnosing FDYT in addition to incongruence/inconsistence. In this context, this large cohort corroborates the existing knowledge and presents sensitivity and specificity figures for a few historical/clinical features that can aid clinicians to establish a positive diagnosis for FDYT.

## Author Roles

(1) Research Project: A. Conception, B. Organization, C. Execution; (2) Statistical Analysis: A. Design, B. Execution, C. Review and Critique; (3) Manuscript Preparation: A. Writing of the First Draft, B. Review and Critique.

T.E.: 1A, 1B, 1C, 2A, 2B, 2C, 3A, 3B

G.D.: 1A, 1B, 1C, 2A, 2B, 2C, 3A, 3B

C.G.: 1A, 1B, 1C, 2A, 2B, 2C, 3A, 3B

E.M.: 1B, 1C, 3B

G.F.: 1B, 1C, 3B

F.B.: 1B, 1C, 3B

A.M.: 1B, 1C, 3B

R.C.: 1B, 1C, 3B

L.M.R.: 1B, 1C, 3B

A.A.: 1B, 1C, 3B

A.P.: 1B, 1C, 3B

M.Z.: 1B, 1C, 3B

M.C.A.: 1B, 1C, 3B

L.M.: 1B, 1C, 3B

M.P.: 1B, 1C, 3B

P.G.: 1B, 1C, 3B

M.M.M.: 1B, 1C, 3B

A.B.: 1A, 1B, 1C, 2A, 2B, 2C, 3A, 3B

M.T.: 1A, 1B, 1C, 2A, 2B, 2C, 3A, 3B

## Disclosures

### Ethical Compliance Statement

The study was approved by the ethical review board of the University of Cagliari (identification no. PG/2018/11698) and the University of Verona, Azienda Ospedaliera Universitaria Integrata Verona (1757CESC) and performed according to the Declaration of Helsinki. The requirement for informed consent was waived because the study was register‐based and no individuals were identifiable at any time. We also confirm that we have read the Journal's position on issues involved in ethical publication and affirm that this work is consistent with those guidelines.

### Funding Sources and Conflicts of Interest

No specific funding was received for this work and the authors declare that there are no conflicts of interest relevant to this work.

### Financial Disclosures for the Previous 12 Months

Roberto Ceravolo reports receiving speaking fees from Zambon, UCB Pharma, AbbVie, and Lusofarmaco. Alberto Albanese reports receiving honoraria from Bial Pharmaceuticals. Maurizio Zibetti reports receiving grant support and speaker honoraria from AbbVie; speaker honoraria from Bial Pharmaceuticals; and travel grants from Merz, Medtronic, Boston Scientific, and UCB Pharma. Maria Concetta Altavista reports receiving consultancy fees as a member of the advisory board for Ipsen (Assago, Milan) and Allergan (Rome). Martina Petracca reports receiving consultancy fees as a member of the advisory board for Zambon Italia s.r.l.

## Supporting information

**Appendix S1.** Names and affiliations of the members of the Italian Registry of Functional Motor Disorders and Italian Registry of Adult Dystonia Study Groups.
