# Supplementary material for: Sudden Onset, Fixed Dystonia and Acute Peripheral Trauma as Diagnostic Clues for Functional Dystonia
Source: Mov Disord Clin Pract. 2021 Sep 10;8(7):1107–11. doi: 10.1002/mdc3.13322 (PMC8485608; doi:10.1002/mdc3.13322)
Supplement: Supplementary file 1 — Appendix S1. Names and affiliations of the members of the Italian Registry of Functional Motor Disorders and Italian Registry of Adult Dystonia Study Groups. [file MDC3-8-1107-s001.docx]

**Supplementary Text S1.**

Gina Ferrazzano MD, PhD^1^, Giuseppe Magro MD^2^, Fabio Bombardieri MD^2^, Sonia Mazzucchi MD^3^, Roberto Eleopra MD^4^, Francesca Valentino MD^5,6^, Brigida Minafra MD^5,6^, Leonardo Lopiano MD, PhD^7^, Carlo Alberto Artusi MD^7^, Luigi Polidori MD^8^, Valentina Durastanti MD, PhD^8^, Anna Rita Bentivoglio MD, PhD^9,10^, Paola Zinzi^9,10^, Carmen Terranova MD, PhD^11^, Amelia Brigandì MD^11^, Marcello Esposito MD, PhD^12^

^1^ Department of Human Neurosciences, Sapienza University of Rome, Viale dell’Università 30, 00185 Rome, Italy.

^2^ Botulinum Toxin Center, Neurology Unit A.O.U. Mater Domini, Catanzaro, Italy.

^3^ Neurology Unit, Department of Clinical and Experimental Medicine, University of Pisa, Italy.

^4^ Parkinson and Movement Disorders Unit, Fondazione IRCCS Istituto Neurologico Carlo Besta, Milan, Italy.

^5^ Department of Brain and Behavioral Sciences, University of Pavia, Italy.

^6^ IRCCS Mondino Foundation, Pavia, Italy.

^7^ Department of Neuroscience - Rita Levi Montalcini, University of Turin, Turin, Italy.

^8^ UOC di Neurologia, Ospedale San Filippo Neri, ASL Roma 1, Roma.

^9^ Fondazione Policlinico Universitario 'Agostino Gemelli' - IRCCS, Rome, Italy.

^10^ Institute of Neurology, Università Cattolica del Sacro Cuore, Rome, Italy.

^11^ Department of Clinical and Experimental Medicine, University of Messina, Italy.

^12^ Clinical Neurophysiology Unit, Cardarelli Hospital, Naples, Italy.
